# 3D Object Detection via 2D Segmentation-Based Computational Integral Imaging Applied to a Real Video

**DOI:** 10.3390/s23094191

**Published:** 2023-04-22

**Authors:** Michael Kadosh, Yitzhak Yitzhaky

**Affiliations:** Department of Electro-Optics and Photonics Engineering, School of Electrical and Computer Engineering, Ben-Gurion University of the Negev, Beer Sheva 84105, Israel; michaelkad77@gmail.com

**Keywords:** computational integral imaging, 3D objects detection, instance segmentation, 3D imaging, depth estimation

## Abstract

This study aims to achieve accurate three-dimensional (3D) localization of multiple objects in a complicated scene using passive imaging. It is challenging, as it requires accurate localization of the objects in all three dimensions given recorded 2D images. An integral imaging system captures the scene from multiple angles and is able to computationally produce blur-based depth information about the objects in the scene. We propose a method to detect and segment objects in a 3D space using integral-imaging data obtained by a video camera array. Using objects’ two-dimensional regions detected via deep learning, we employ local computational integral imaging in detected objects’ depth tubes to estimate the depth positions of the objects along the viewing axis. This method analyzes object-based blurring characteristics in the 3D environment efficiently. Our camera array produces an array of multiple-view videos of the scene, called elemental videos. Thus, the proposed 3D object detection applied to the video frames allows for 3D tracking of the objects with knowledge of their depth positions along the video. Results show successful 3D object detection with depth localization in a real-life scene based on passive integral imaging. Such outcomes have not been obtained in previous studies using integral imaging; mainly, the proposed method outperforms them in its ability to detect the depth locations of objects that are in close proximity to each other, regardless of the object size. This study may contribute when robust 3D object localization is desired with passive imaging, but it requires a camera or lens array imaging apparatus.

## 1. Introduction

3D object detection and segmentation can be useful in various fields, such as autonomous vehicles [1], robotic navigation [2], medicine [3], and surveillance [4]. In these cases, the motivation is to enable 3D tracking of objects in the scene. The advantage of 3D with regard to traditional two-dimensional (2D) imaging techniques is its capability to capture depth information of different objects that are in a scene. In recent studies, it was sown that depth-based object isolation may improve prosthetic vision [5,6].

Integral-Imaging [7,8] is a 3D passive imaging technique, which can be realized using a multi-channel camera array, where each camera records a 2D image from a slightly different angular perspective of the scene. These images are referred to as Elemental Images (EIs). 3D data can be digitally reconstructed from these multiple-perspective images using Computational Integral Imaging (CII) [8,9,10,11,12,13,14,15,16]. While Integral-Imaging can produce rich 3D data of the scene, it does not require the use of sources of illumination, as in time-of-flight cameras [17] or structured light imaging [18]. Compared to conventional stereo imaging, it does not require complex measurements as may be needed for disparity calculations [19], and it is more robust in very noisy imaging conditions [20] compared to conventional stereo-vision. A recent study showed that passive integral imaging provides better image reconstruction compared to long-wave infrared (LWIR) cameras and LiDAR (time of flight) imaging systems using Azure Kinect RGBD cameras in degraded environments such as fog and partial occlusion [21].

In this study, we performed 3D object localization by producing the depth locations of the objects in a 2D image detected via deep learning, using integral-imaging data. We exploit depth-based blurriness characteristics of such data, which can enable accurate depth localization. Such an approach does not require the use of scene illumination as in active imaging, while, on the other hand, it efficiently uses CII depth-based properties that may be more robust than depth cues in a single image or in stereo imaging, as stated above.

The rest of this paper is organized as follows. The next two sub-sections present previous related works on 3D object detection using integral imaging and the proposed contribution. Section 2 gives an overview of the previously-developed technique of CII for 3D data formation using integral imaging and our previous approach (that we partly use here) for locating depths regions where large objects may exist, using the CII data. Section 3 describes the proposed method for 3D of object detection. Section 4 presents results for a realistic 3D scene, and Section 5 concludes.

### 1.1. Previous Related Works

Earlier methods based on integral imaging [22,23,24] automatically find only the object depth location and use the elemental image (EI) as a reference image. In [22], the depth is estimated by correlating the reconstructed images to the EI at each pixel location. This method is computationally heavy and may have false detections in smooth regions. Another method [23] makes a comparison between the reconstructed images and the EI and looks for the minimum standard deviation in order to find the 3D object’s depth position. This method would not be effective in cases that include objects at multiple depth planes and non-uniform backgrounds. In [24], block-matching is used for finding the minimum standard deviation inside a block instead of the whole EI. While this method can fit the case of objects at multiple depth planes, the size of the block is chosen manually, and the results are sensitive to this size. In [25], depth estimation is found according to the focus characteristic by evaluating the Laplacian in the refocused image, in addition to the comparison to the EI. It is simple but computationally heavy. These methods perform depth estimation but not 3D object detection and isolation. Ref. [26] performs a 3D object isolation process that is based on the minimum variance between the reconstructed images and the EI. This method may perform well in regular illumination conditions, but it would be less effective under very noisy conditions, and similar-depth objects cannot be isolated separately.

In [27,28], a method for 3D object localization and isolation using computational integral imaging was proposed. The main idea was to capture the 3D scene from multiple view perspectives obtained by shifting a camera in fixed intervals and recording an image at each step to form an array of elemental images. Then, reconstructed depth planes were calculated using computational integral imaging (detailed in Section 2). Since an object at a certain depth will be sharp only at the reconstructed plane of the same depth. Thus, by calculating the average gradient magnitude of each reconstructed plane, the depth locations of objects can be obtained according to the depths where the peak values in the average magnitude appear. This method used a threshold to isolate the objects in the reconstructed image gradient. However, it is difficult to obtain the appropriate threshold since it may change for different cases due to illumination, the complexity of the scene etc. Moreover, with a threshold alone, adjacent objects may not be isolated as separate objects. In addition, small objects or objects located in adjacent planes of larger objects may not be detected by the average gradient magnitude of their reconstructed plane since they have a small amount of gradient energy relative to the larger objects in adjacent planes.

In a recent study [29], a deep learning integral imaging system was proposed that can reconstruct a 3D object without dealing with the out-of-focus (blurred) areas that occur in the Integral-Imaging computationally reconstructed depth planes. Targets in the scene are first detected and segmented in the 2D elemental images using a pre-trained Mask R-CNN. Then, the depth-plane reconstruction is performed only at the segmented object regions, while blurred regions are removed (have zero value). However, the depth locations of the objects are not found automatically in this process, and prior information on them should be obtained when performing the imaging. 

### 1.2. The Proposed Contribution

Unlike the previous works, here we achieve passive 3D object detection by exploiting both scene 2D content recognition via machine learning and physical blur-sharpness depth-based properties inherent to CII. Differently from the method in [25,26], the proposed method does not rely on a threshold parameter for object depth recognition and performs much better with small objects and the same or similar-depth objects. Unlike the method in [27], which calculates reconstructed depth planes only at the regions of the detected objects using computational integral imaging for known depths of the detected objects. Our method finds the depth locations of the detected objects automatically without prior knowledge of the depth positions. The method first applies 2D detection and segmentation of objects in the scene using deep learning instance segmentation and classification over a recorded elemental image. We used, for that, a pre-trained Mask R-CNN algorithm. Depth locations of the objects are then detected using CII blur-based analysis at each object’s depth tube. In this approach, the object depth localization operation would likely not be influenced by nearby objects, and it will be considerably more robust than examining the whole reconstructed plane to localize objects, as done previously [28,30].

Furthermore, we used in this study a new integral-imaging camera-array device developed recently by our group [31]. This camera array can simultaneously capture an array of videos, a property that enables us to perform dynamic object tracking in a 3D space with continuous tracking of the objects’ depths.

## 2. Computational Integral Imaging (CII) Analysis for Depth Data Formation

**Figure 1:**
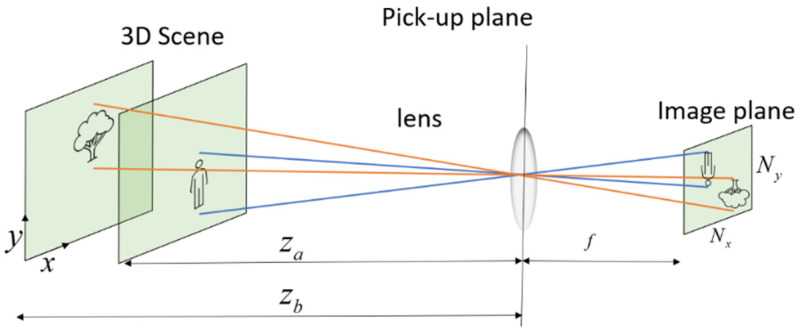
A single camera imaging illustration and parameters, where objects in a 3D space are imaged into the image forming an elemental image or video.

**Figure 2:**
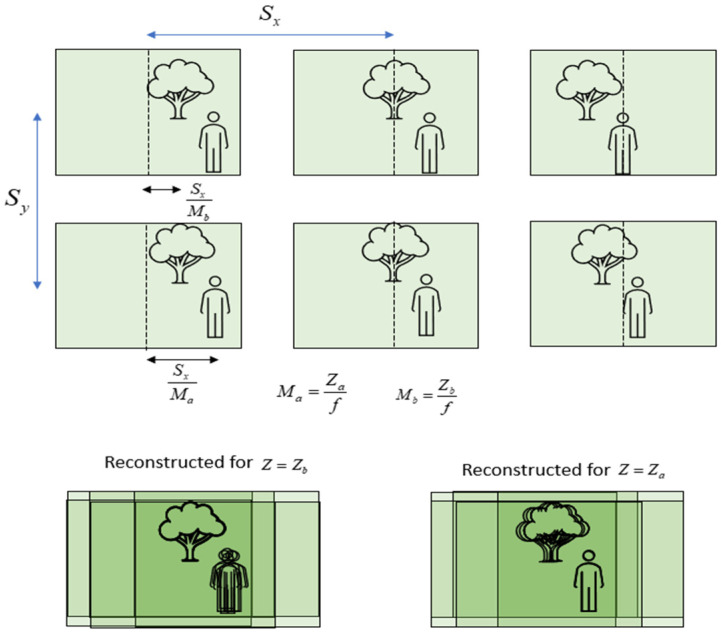
An illustration of the CII process (Equation (1)) and its parameters. Six EIs (**upper part**) are summed according to Equation (1) onto two virtual depth planes, one at the depth of the tree, $z_{b}$, where the tree is imaged sharply (**lower-left**), and the other at the depth of the person, $z_{a}$, where the person is imaged sharply (**lower-right**). Objects at other depths become blurry.

In integral imaging, 3-D object reconstruction can be performed either optically or computationally. Computational reconstruction typically mimics optical reconstruction; however, it has the additional flexibility of digitally manipulating the data to extract better visual information [7]. The reconstructed depth plane of the integral imaging system at $z_{0}$ depth for an array of EIs is [8,32]:

(1) $$f^{RP}(x,y,z_{0})=\frac{1}{KL}\sum_{k=0}^{K-1}\sum_{l=0}^{L-1}g_{k,l}\left(x+\left(\frac{1}{M_{z_{0}}}\right)S_{x}k,y+\left(\frac{1}{M_{z_{0}}}\right)S_{y}l\right)$$

where $g_{k,l}$ is an elemental image with k and l indices, K×L are the number of EIs in the array, and M is the magnification factor that the ratio between the distance from the camera to the reconstructed plane and the camera’s focal distance. $S_{x}$ and $S_{y}$ are the distances between the cameras in the x and y directions (x and y define the camera’s plane), respectively, and $f^{RP}(x,y,z_{0})$ is the 2D reconstructed plane at a distance $z_{0}$ from the camera. Figure 1 presents an illustration of the optical path, which demonstrates single-camera imaging. Figure 2 illustrates the CII process according to Equation (1). The upper part of the figure shows an illustration of 6 EIs that present a scene from slightly different angular perspectives, while the lower part illustrates reconstructed planes (Equation (1)) at the depths of the tree, $z_{b}$ (lower left, where the tree is sharp), and the person, $z_{a}$ (lower right, where the person is sharp). It can be seen that, at the depth of the tree, multiple shifted images of the person (from different EIs) are summed and create blurriness in the reconstructed frame. The same is demonstrated in the reconstructed plane at the depth of the person. As the number of EIs increases, the appearance of multiple objects becomes more blurry, and this may affect the ability to distinguish sharp from blurred regions [30].

The quality of the synthesized image using computational reconstruction is better than that of the images reconstructed optically [8]. The computational reconstruction itself is free of diffraction and device limitations, however, each camera in the array has physical device limitations, and misalignment between cameras may occur. These inaccuracies can be accounted for computationally [31].

The ability of the computational integral imaging system to separate between two adjacent depth planes is limited. This limitation is called depth resolution or longitude resolution, and it defines the minimum step between reconstructed planes that produces a shift of one pixel in the camera sensor [32].

### Locating Depths Where Large Objects Exist in the Reconstructed Planes

As stated briefly in Section 1.1, the previous works section above, the depth locations of objects were found by comparing the average gradient magnitude of each reconstructed plane [27,28]. Since objects which are originally located at the reconstructed plane depth are reconstructed properly and in focus (sharp) while other objects become blurred, the average gradient at this depth will likely have a higher value because sharp regions have higher gradient magnitudes. The Average Gradient Magnitude of a Reconstructed plane (AGMR) at depth z is:

(2) $$AGMR(z)=\frac{1}{N_{x}N_{y}}\sum_{y}\sum_{x}\left|\nabla \left(f^{RP}(x,y,z)\right)\right|,$$

where $N_{x}$ and $N_{y}$ are the numbers of pixels along the *x* and *y* directions, respectively, and ∇ is the gradient magnitude operator. Plotting the average gradient magnitude values against the depth locations on a graph will produce local maxima in depths that include large focused regions, which may belong to relatively large objects at these depths.

The separation between blurred areas and the objects that appear sharp in the reconstructed depth plane was performed by a threshold over the gradient magnitude of the reconstructed plane at the depth found by the peak of the AGMR [28]. However, this approach, in this form, struggles to perform well in cases of small objects or when objects are adjacent to each other at near-depth planes. Another difficulty is the setting of the threshold value. 

## 3. Proposed Method for 3D Object Detection and Segmentation

**Figure 3:**
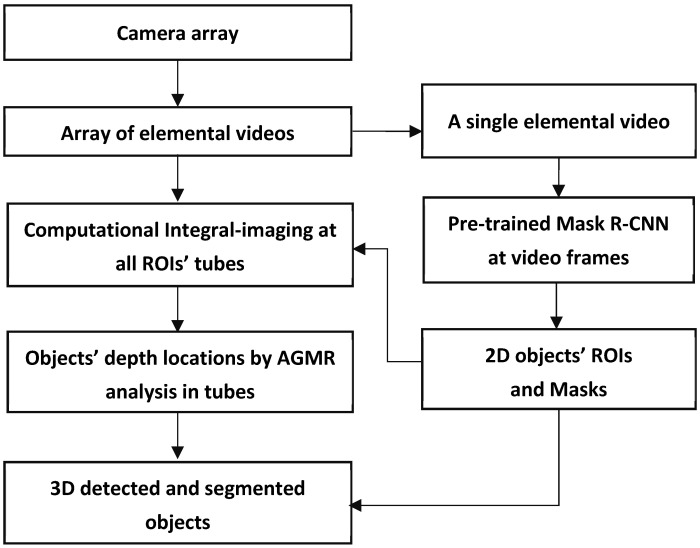
A scheme of the whole 3D object detection and segmentation process.

An overall description of the proposed method is schematized in Figure 3. In short, a camera array creates an array of images or videos, termed Elemental Videos (EVs), where each image or video observes a slightly different angular perspective of the scene. At each time instance, the array of frames of these videos constitutes the current Elemental Images (EIs), which can also be termed Elemental Frames. Object detection using deep-learning-based instance segmentation is applied to a central elemental image in the video, producing regions (bounding boxes) and masks of the detected objects in the 2D image of the 3D scene. Each of the 2D detected objects at the current video frame goes through a local computational integral imaging at its bounding box region, forming a reconstructed depth tube constructed of local depth planes. All of the detected local objects’ tubes go through local AGMR computations that give the depth locations of the 2D detected objects, producing 3D object detections. Below is a detailed description of the method.

### 3.1. Capturing Elementals Videos (EVs) with a Camera Array

**Figure 4:**
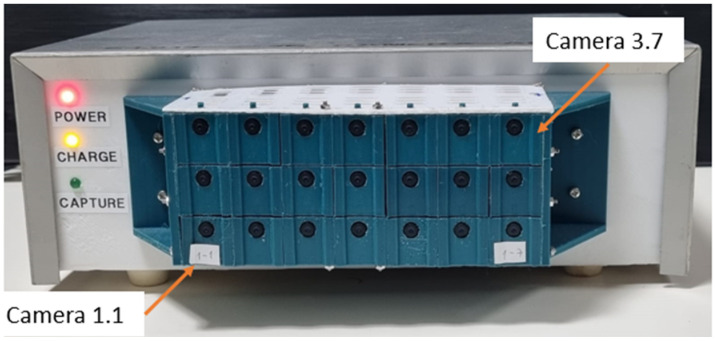
The array imaging system with 7 × 3 mini cameras used for computational integral imaging [31].

To capture the elemental videos, we used a new system developed by our group [31]. The imaging system consists of 21 simple small cameras arranged in the form of a matrix of 3 rows by 7 columns (Figure 4). The small cameras employed were the SQ11 mini-HD [33]. Each camera has a digital resolution of 4032 × 3024 for image capture and 1280 × 720 pixels or 1920 × 1080 pixels for video capture, a frame rate of 15 or 30 frames per second, and a viewing angle of 140°. The camera’s focal distance, which was used for the calculation of the magnification, as shown in Figure 1, is 10 mm. The horizontal and vertical distances between each camera are 21.1 mm. The system is controlled by computer software and allows both still and video photography simultaneously by all the array cameras. 

**Figure 5:**
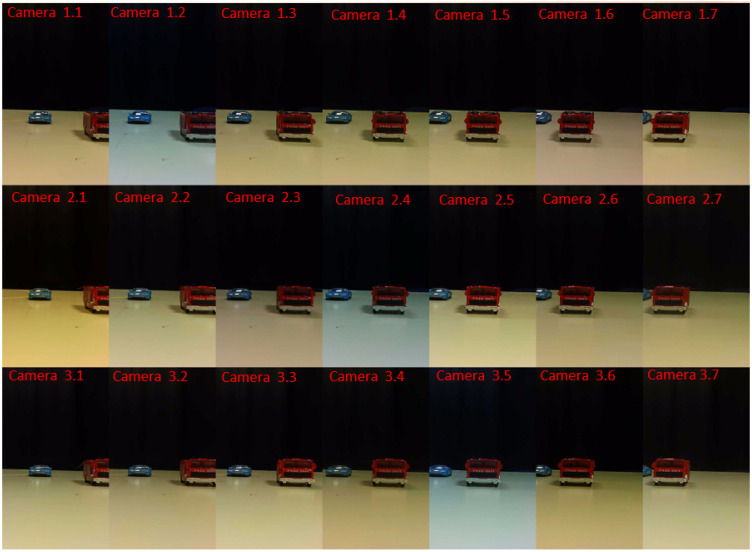
An example of a camera array output video frame-array (elemental frames). The video includes a static blue toy car 30 cm away from the imaging system and a red track moving away from the system, starting at 20 cm from the system. The elemental videos can be seen at https://www.youtube.com/watch?v=LYWr04gMUCY (accessed on 20 February 2023).

Since we used for the camera array prototype, low-cost cameras and not sufficiently accurate array construction needed for the computational integral imaging operation, we applied a process of aligning and calibrating the cameras in both axes, horizontal and vertical [31]. In the first stage, at which we calibrated each of the array cameras, we used well-known camera calibration tools [34,35] to find the intrinsic and extrinsic parameters of the cameras and to remove the lens radial distortion. For this goal, we used a chessboard as our calibration target by knowing the exact square size of the board. Next, we found the transformation matrix between the array output images, relative to a reference middle camera output image by finding the matching features between each of the cameras relative to the reference, and relative to them, estimate the 2D affine geometric transformation between the array cameras and the center camera to compare scale, translation, rotation, and shearing. Then, to perform alignment validation in each of the cameras, a robust and flexible visual fiducial marker called AprilTag, which uses a 2D bar code style “tag,” was detected [34]. The AprilTag allows a full 6 degrees of freedom localization of features from a single image. In the last step, each elemental video shifted in a precise way according to the physical shift between the cameras in the camera array, using the image cropping method and a calculated digital offset. Following this procedure, the output of the calibrated camera array is a matrix of 2D-aligned elemental images or videos, each capturing the scene from a slightly different angular perspective. An example of a camera array output for a simple scene of 2 toy vehicles is shown in Figure 5. This is a video scene in which the blue car is static, and the red track is moving away from the camera (a link to the video is provided below the figure).

The vertical field of view of the cameras is large, while the objects in the scene were small enough to fit within the small horizontal field of view imposed by the camera setup. The scene shown in these figures was intentionally kept simple, without a complicated background, for the purpose of visually demonstrating step-by-step the process of the proposed method.

### 3.2. 2D Object Detection via Instance Segmentation

**Figure 6:**
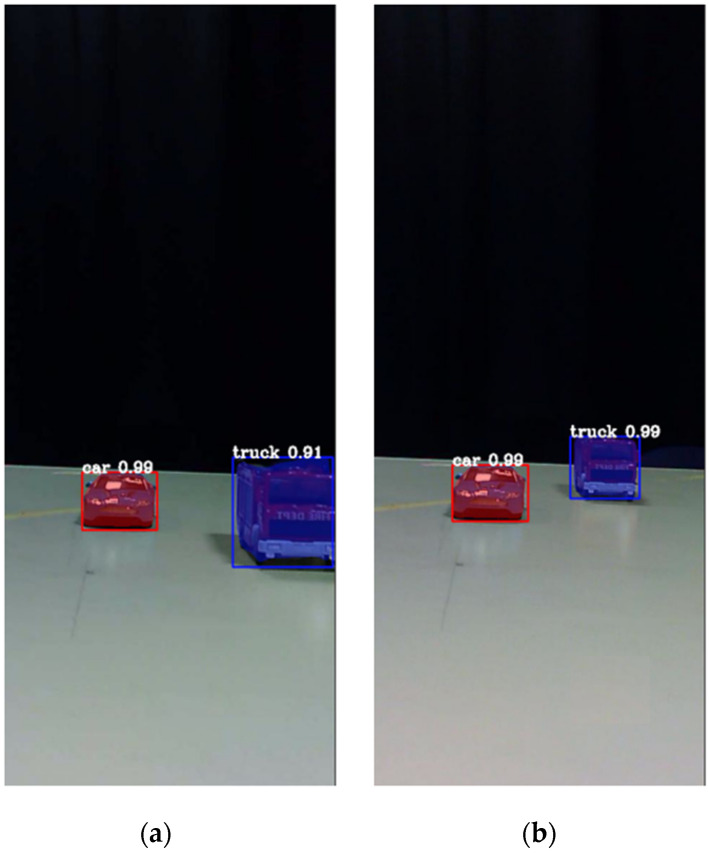
The output of the Mask R-CNN for the selected frames. (**a**) Frame 30, and (**b**) Frame 120. Bounding boxes, masks and classification probabilities are shown for each detected object.

2D object detection is applied to locate objects in the scene. This process produces bounding boxes of the detected objects and also masks that are the pixel locations of the objects. We applied a pre-trained Mask R-CNN [36] trained with a public dataset set (MS Coco dataset [37]) that has 81 classes for 2D object detection and segmentation. The Mask R-CNN algorithm produces the region of interest (ROI) and the pixel mask for each detected object. The Mask-RCNN method was chosen due to its known high accuracy in object detection and segmentation and its ability to efficiently process images in a scene. This makes it a suitable choice for the proposed algorithm and provides more accurate and reliable results. However, other methods such as the YOLACT [38], SOLOv2 [39] or other instance segmentation methods [40] can be applied as well, as initial 2D object detectors for the depth localization process since they also generate ROIs and pixel masks for all the detected objects. When using a pre-trained network, the possible detected objects will be those that belong to categories that the network was trained upon. If more object categories are required, network training should be done in accordance. Figure 6 presents an example of 2 frames of an EV, frame 30 and frame 120, after instance segmentation by the Mask R-CNN. In the image at the left side, the red truck is closer to the camera relative to the blue car, while in the right image the truck is further than the car. As can be seen in Figure 6, the segmentation produces a bounding box for each of the detected objects and also a mask covering the detected object’s region. 

The detected bounding box object regions are used in the next stage to reveal their depth locations.

### 3.3. Finding Objects’ Depth Locations via CII Analysis in Objects’ Tubes

**Figure 7:**
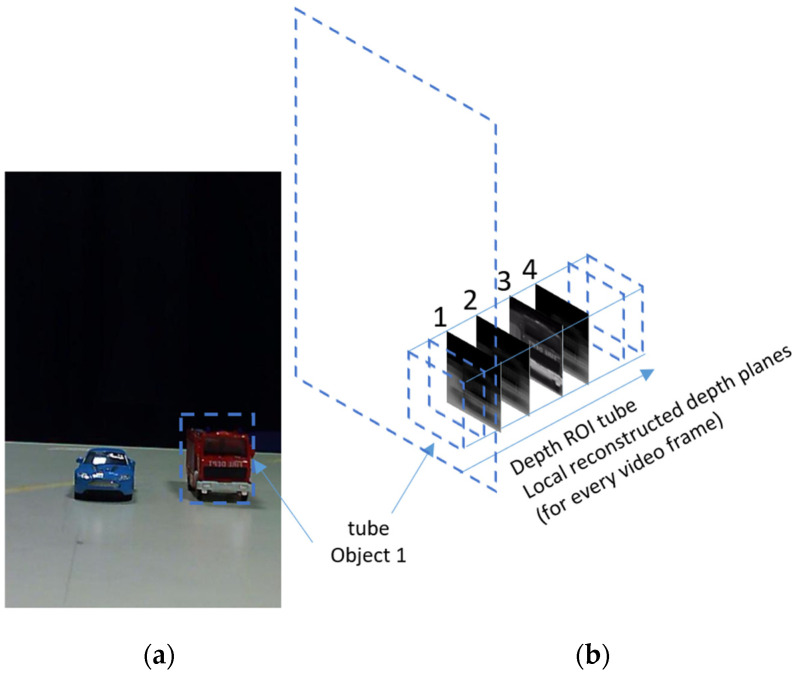
(**a**) An elemental image (one of the images of Figure 2) with the bounding box of a detected truck object. (**b**) A depth ROI tube of the truck consists of locally reconstructed local depth planes. The sharpest local depth plane (numbered 3) is at the depth location of the truck. Values in the graph are calculated in cations of all detected objects regardless of their proximity to each otherופית.

As explained in Section 2, The CII process produces a series of reconstructed depth planes of the 3D scene. An object at a certain depth location will appear sharp at the depth plane associated with its true depth and will get blurrier at other depth planes while moving away from that plane. As stated above, in a previous study, we showed that depth planes with a large number of sharp regions could be found based on a measure of the average gradient (AGMR) over the whole depth plane (Equation (2)). The problem with this is that a small object may not cause a sufficiently strong AGMR over the whole depth plane where it is present, particularly if a larger object exists somewhere not far at a different depth. This also means that same-depth objects cannot be distinguished, and the number of objects that can be detected at different depths is limited as they should be far enough from each other. Here we solve these problems by performing a local AGMR analysis only at object tubes (instead of the entire depth plane). In other words, for each 2D detected object, we have a tube of reconstructed regional depth planes (at the object’s bounding box, along depth), as demonstrated in Figure 7. Figure 7b shows several local reconstructed planes at the depth tube of the detected truck shown in Figure 7a. The sharpest local depth plane (numbered 3) is at the depth location of the truck.

For each object depth tube, the average gradient magnitudes of the local depth planes are calculated (Equation (3)). The depth at which the average gradient of the reconstructed regional depth plane is maximum is the depth location of the object (Equation (4)). With this approach, the calculation for each object is robust, also for small objects, and it is not affected by nearby objects or backgrounds. Additionally, objects at the same depth will be detected separately. Note that in a dynamic scene with moving objects or cameras, the tubes’ locations and sizes can change according to the changing locations and sizes of the objects along the video frames. Thus, the AGMR that was presented in Equation (2) for representing the average gradient magnitude of a whole reconstructed depth plane is modified here for representing the average gradient magnitude of the reconstructed plane $f_{k}^{RP}$ only at a local object ROI (tube) of each detected object in a video frame *k*:

(3) $$AGMR_{k}^{O_{i}}(z)=\frac{1}{N_{x}N_{y}}\sum_{y\in ROI_{i}}\sum_{x\in ROI_{i}}\left|\nabla \left(f_{k}^{RP}(x,y,z)\right)\right|,$$

where *z* is the depth index (distance from the camera) of the reconstructed planes $f_{k}^{RP}$ in the object’s tube (4 local planes are demonstrated in Figure 7b), $O_{i}$ represents the object, *i,* in the frame, and $N_{x}$ and $N_{y}$ are the numbers of pixels along the *x* and *y* directions of the detected bounding box $ROI_{i}$ of the object $O_{i}$. 

The object depth location, $z_{k}^{O_{i}}$, is then the depth, *z,* of the sharpest reconstructed plane of object $O_{i}$ in frame *k*. This plane has the maximum gradient magnitude across the local depth planes in the object’s tube:

(4) $$z_{k}^{O_{i}}=\arg\max\left(AGMR_{k}^{O_{i}}\left(z\right)\right)$$

**Figure 8:**
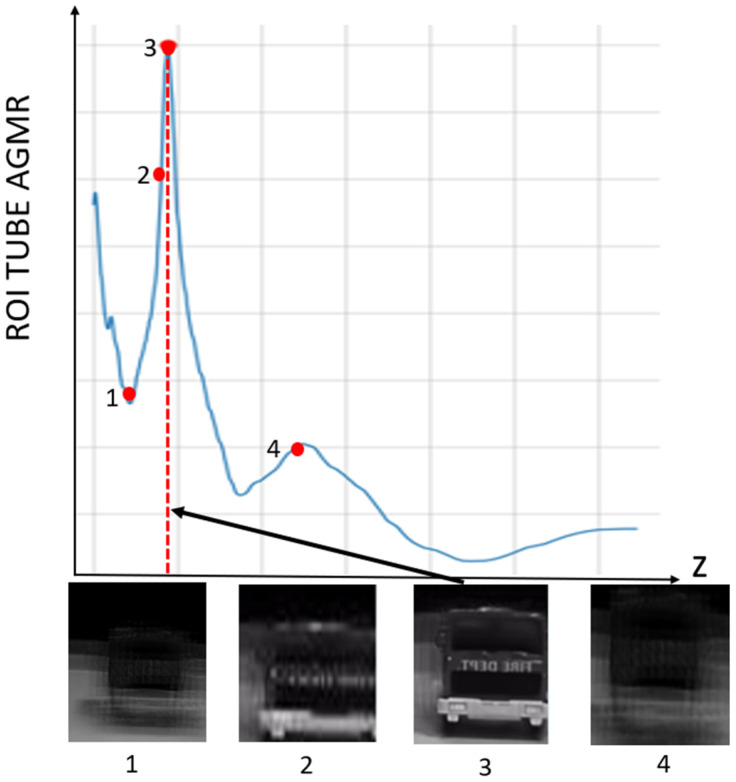
ROI tube AGMR of the detected truck object, plotted against depth, *z*. The values in the graph are calculated in Equation (3). The highest peak is obtained for the most focused local reconstruct depth plane, where the object is located (Equation (4)). Below are four locally reconstructed depth planes, where the third is the sharpest, indicated by the highest peak.

Figure 8 presents the AGMR graph (Equation (3)) for the detected truck object. The maximum in the graph is the depth location of the object, $z_{k}^{O_{i}}$ (Equation (4)), associated with the sharpest local reconstructed depth plane (numbered 3 in the bottom part of the figure).

**Figure 9:**
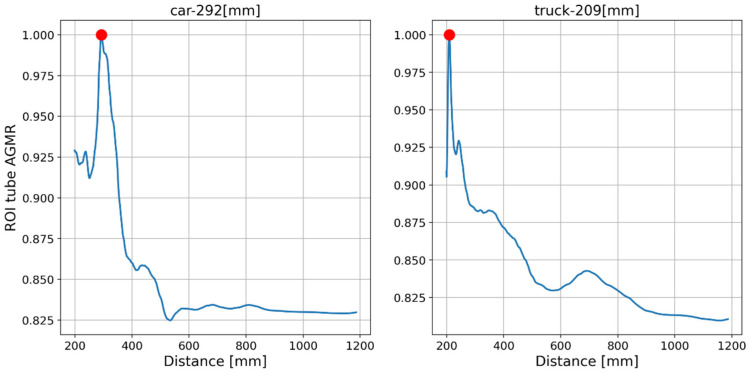
AGMR graphs in objects’ tubes (Equation (3)) for frame 30. The red dot point on the highest gradient value is received for the sharpest plane in the tube.

**Figure 10:**
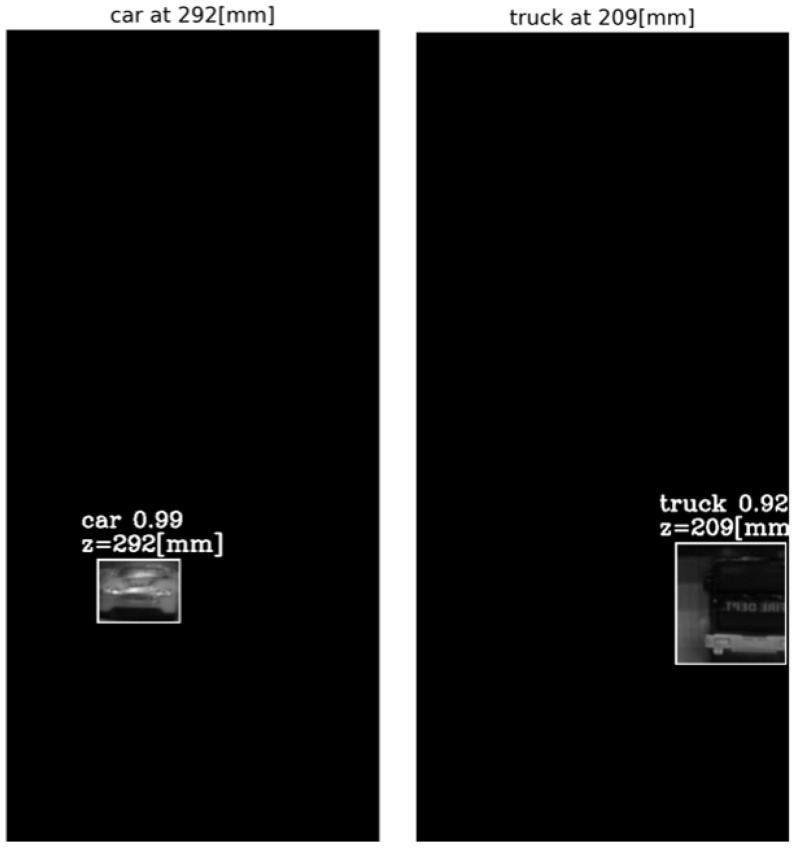
The reconstructed isolated region for each detected object in frame 30. The spatial location is according to Mask R-CNN, and the depth location is according to the peak in the object’s ROI tube AGMR (Figure 9).

**Figure 11:**
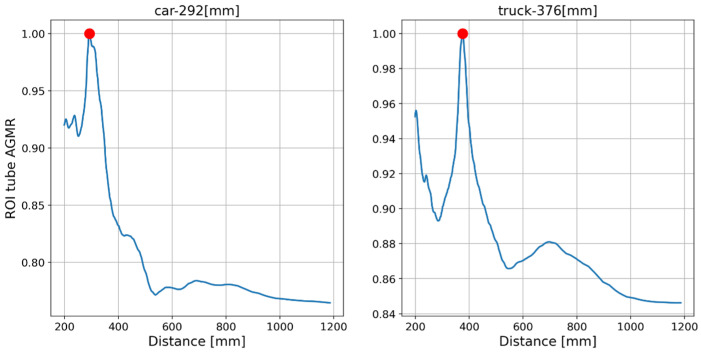
The AGMR in objects’ tubes (Equation (3)) for frame 120. The car is in the same depth while the truck moves backward, as expected.

**Figure 12:**
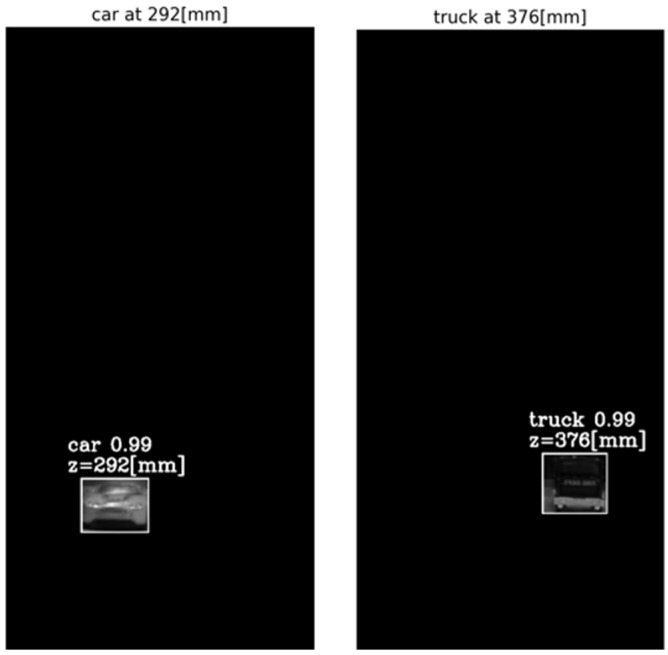
Same as Figure 10, but for frame 120. Here each depth location is according to the peak in the object’s ROI tube AGMR in Figure 11.

The output of the method for a video frame includes the bounding boxes and masks of all the detected objects, their classes and prediction accuracy, and their depth locations according to the peaks of their own tube AGMR graphs. Figure 9 demonstrates AGMR graphs of the tubes of the detected car and truck objects in frame 30. The peaks indicate the objects’ depth locations. Figure 10 presents the output of the method for this frame. Figure 11 and Figure 12 present the same as Figure 9 and Figure 10, but for video frame 120, at which the truck is further than the car.

It can be seen that the static toy car was detected at the same depth in both frames, while the moving toy truck was detected at depth according to its current 3D location in each frame. In all cases, a sharp peak in the local tube AGMR indicates the object’s depth. In addition, it can be seen that even though there is a partial occlusion of the car, meaning that in several cameras, the car is partially visible (Figure 5), the algorithm managed to computationally reconstruct the object and produce the depth location. 

## 4. Experimental Results

This section presents the results of the implementation of the proposed method for a real-life scenario. The method is evaluated by its ability to find the depth locations of all the objects detected in a 2D elemental image of the scene. 

**Figure 13:**
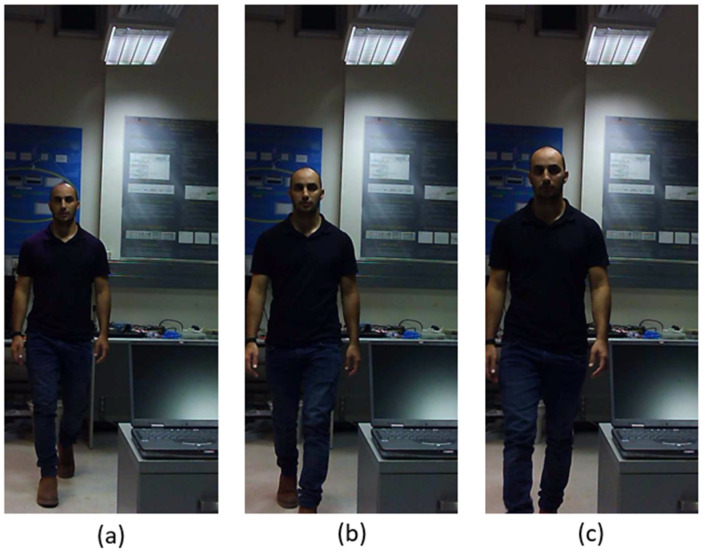
Three elemental frames from a real-life video, at which a person moves toward the camera array approaching a laptop computer located at a fixed distance of about 1.3 m, and then moves away. In (**a**–**c**), the moving person is at distances of about 4.7 m, 3.2 m and 1.6 m from the camera, respectively. [https://www.youtube.com/watch?v=maBp7F_8QwE (accessed on 20 February 2023)].

An example of a scene with real size daily objects and with non-uniform background is presented here. The scene is indoor and includes a person that starts moving at about 5 m away from the camera array approaching a laptop computer located at a fixed distance of about 1.3 m from the system and then moves back. The duration of the video is 5 s (three sample frames are shown in Figure 13).

**Figure 14:**
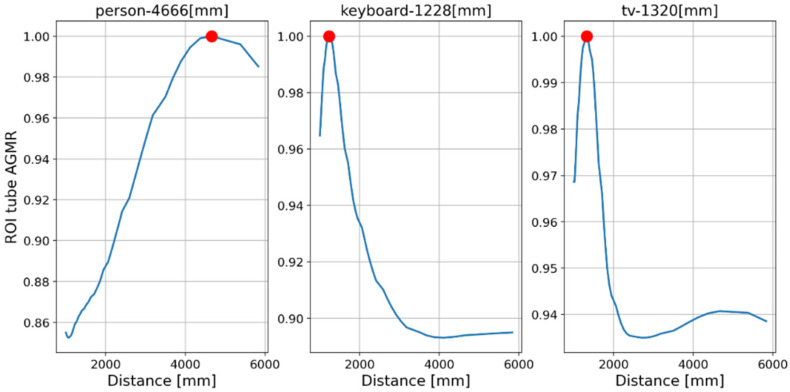
Tube AGMR graphs for the first video frame (Figure 13a) calculated at the three detected objects’ tubes (Equation (3)). The horizontal axis is the distance from the camera. The three objects detected in this frame by the Mask R-CNN are a person, a keyboard and a TV. The peaks in the graphs indicate their assessed depth locations.

**Figure 15:**
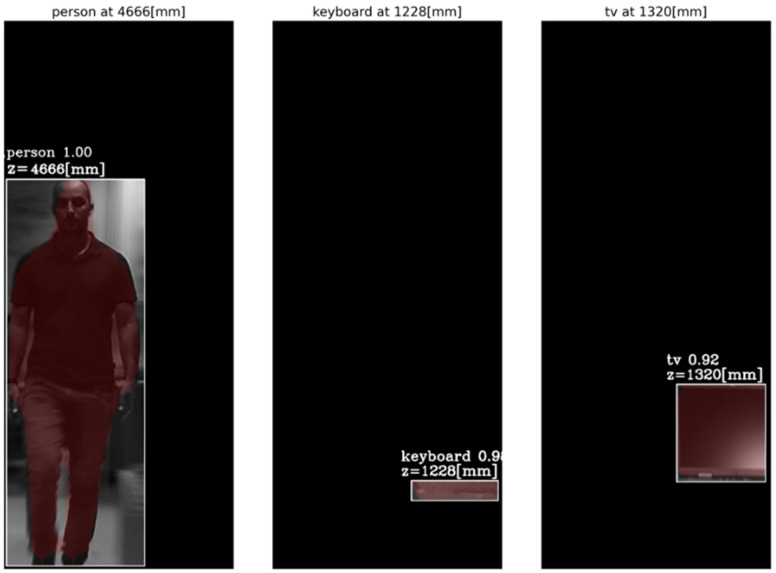
The reconstructed isolated depth plane for each object in the first sampled frame (Figure 13a). The spatial location, the class (with recognition probability) and the brown mask are according to the Mask R-CNN, while the depth location, z, is according to the ROI tube AGMR peak (Figure 14).

First, objects were detected by the Mask R-CNN in the frames of a single central elemental video. In these frames, the detected categories by the Mask R-CNN were a person, a keyboard and a TV screen (a false classification of a laptop screen). Figure 14 presents the AGMR graphs for the first of the three frames (Figure 13a), calculated at the tubes of the three detected objects (Equation (3)), with peaks that indicate their depth locations (Equation (4)). Figure 15 presents results for this frame, which include the reconstructed isolated object for each detected object (that is, the local depth plane at the peak of its graph), with its depth location and predicted category written above. According to Figure 14, the first depth location of the person in Figure 13 is 4666 mm from the camera array system, the keyboard is located at 1228 mm, and the computer screen that is classified as a TV is located at 1320 mm.

**Figure 16:**
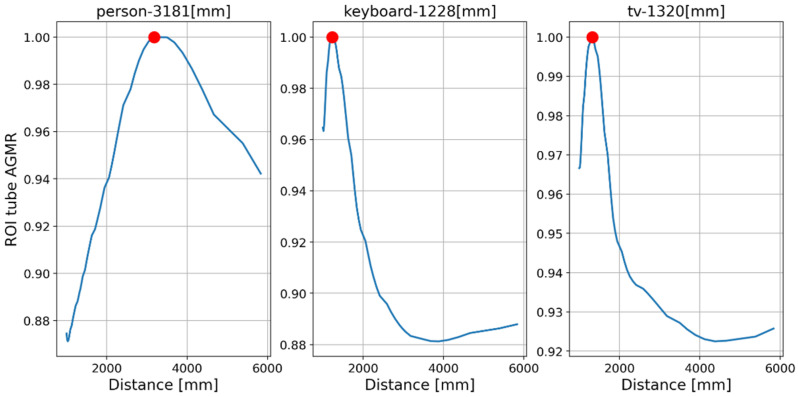
Same as Figure 14, but for the second sample video frame (Figure 13b). Note that based on the locations of the peaks, the moving person here is closer to the camera, while the locations of the static objects are the same.

**Figure 17:**
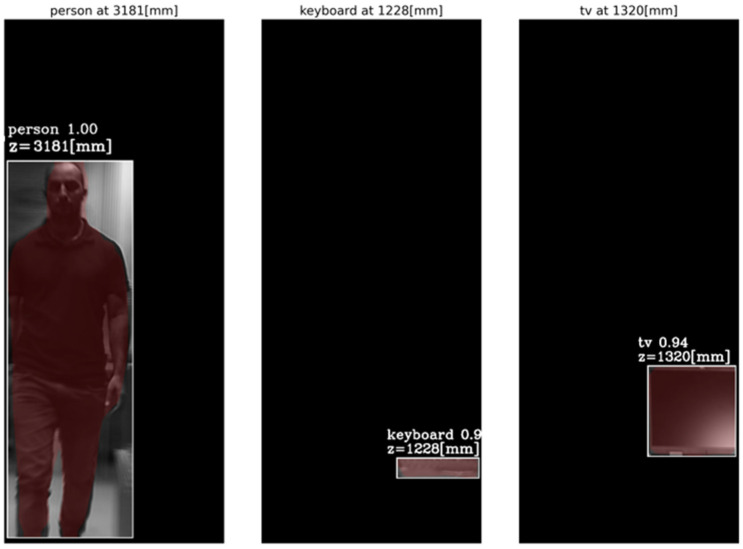
Same as Figure 15, but for the second sampled frame (Figure 13b). Here the detected objects’ depth locations are according to Figure 16.

**Figure 18:**
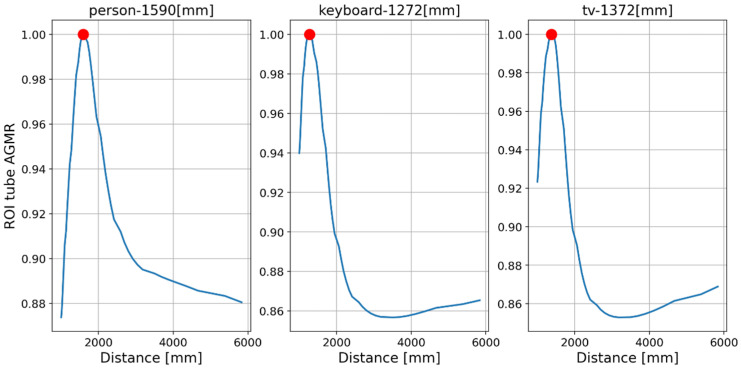
Same as Figure 14, but for the third sample video frame (Figure 13).

**Figure 19:**
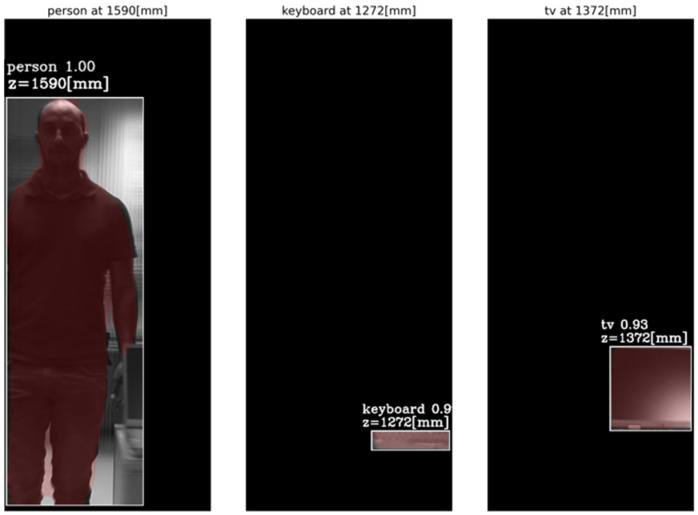
Same as Figure 15, but for the third sampled frame (Figure 13c). Here the detected objects’ depth locations are according to Figure 19. Note that the locations found for the static objects in this frame are about 5 cm different from their correct depths found in the previous frames.

Figure 16 and Figure 17 present the tube AGMR graphs and the corresponding 3D detected objects for the second video frame example (Figure 13b). The same is shown in Figure 18 and Figure 19 for the third video frame example (Figure 13c). A video output of the method (AGMR graph and the object detection) for the moving person can be seen at https://www.youtube.com/watch?v=maBp7F_8QwE (accessed on 20 February 2023).

From the results obtained (Figure 14, Figure 15, Figure 16, Figure 17, Figure 18 and Figure 19), the algorithm detects the movement of the person toward the computer, and for each frame, the depth locations of all the objects are obtained. In addition, even though some of the category predictions obtained by the Mask R-CNN are false (i.e., a computer screen classified as a TV), the 3D object detections, which are the goal of this study, are performed well.

### Comparison with the Previous Method

**Figure 20:**
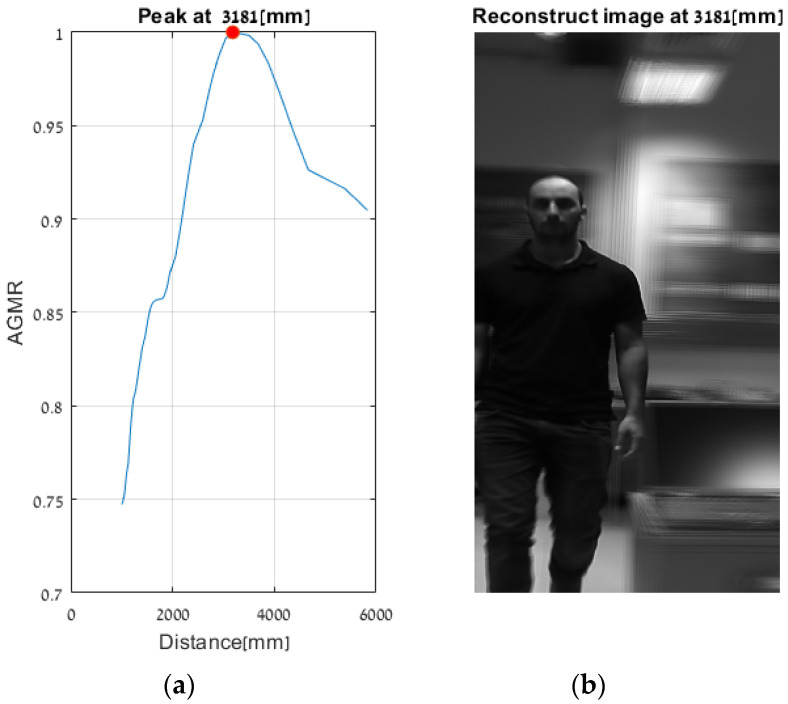
Applying the method in [28] to the video frame example in Figure 13b. (**a**) An AGMR graph at which each point is the average gradient magnitude of the whole reconstructed plane at each distance from the camera (Equation (1)). The peak indicates the depth location of the larger (person) object, while other objects, such as the laptop, are not detected (compared to Figure 16, where depths were found for all the objects detected by the Mask R-CNN). (**b**) The reconstructed plane at the peak of the AGMR shows a sharp image of a person.

**Figure 21:**
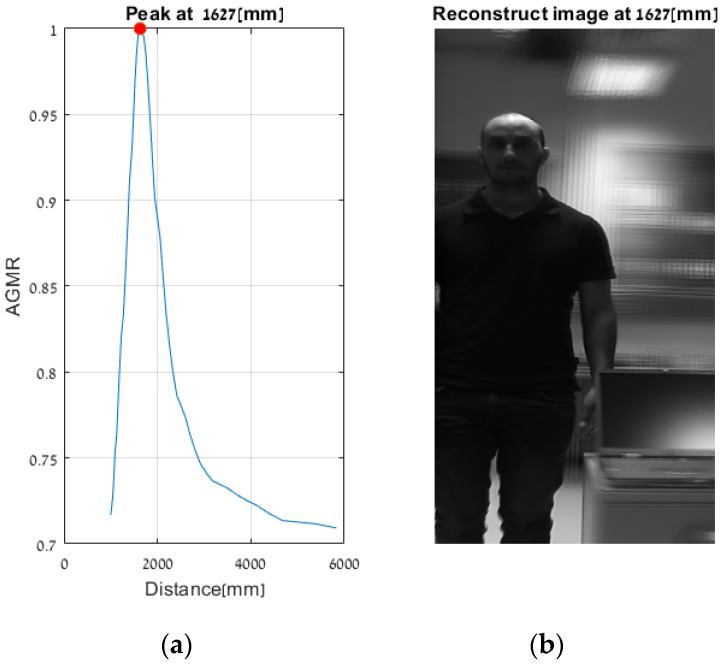
Same as Figure 19 but for Figure 13c. Here the person is very close to the laptop (about 22 cm), and the method in [28] obtained a single sharp peak that represents all objects around the same distance. At the same time, the proposed method found the depth locations of all detected objects regardless of their proximity to each other, as can be seen in Figure 18.

As explained in Section 1.1, in a previous study, we applied an AGMR to the whole reconstructed plane, i.e., not at detected object tubes and without the use of a preliminary 2D object detection. Thus, a single AGMR graph is produced for each recorded image array (unlike multiple graphs according to the number of detected objects). Therefore, instead of a single pick at the depth of the object, the single AGMR graph may have several picks at several different objects’ depths. Objects were extracted based only on the blur-sharpness properties in the reconstructed depth planes. A peak in the AGMR indicates a reconstructed plane of depth with significant sharp regions, which means an existing object at that depth. Examples of applying this method to two of the recorded video frames (Figure 13b,c) are shown in Figure 20 and Figure 21. The difference between the cases is the depth distance between the person and the laptop objects (about 90 and 22 cm, respectively). We can see that in both cases, the method in [28] produced only one peak in the AGMR graph. This means that only a single object was detected in the frame. The reconstructed planes at that peak depths are shown in Figure 20b and Figure 21b. In both cases, the sharp person is the detected object, while the laptop could not create its own peak. In Figure 21b, the laptop is only mildly blurred as its depth location is close to the person. Compared to these results, the proposed method in Figure 16 and Figure 18 clearly found the depth locations of all the detected objects. In this case, each object produces its own AGMR graph, and the method is not sensitive to the object size or to the number of objects in the scene. 

## 5. Conclusions

In this paper, we proposed a novel method for detecting objects in a 3D space via integral imaging, where depth locations of the detected objects in the scene are found with high accuracy and without using active imaging. 

We used a newly developed camera array of 7 × 3 mini cameras that simultaneously record an array of images or videos, where each image or video observes a slightly different angular perspective of the scene. We applied 2D detection and segmentation of objects in the scene using a pre-trained Mask R-CNN. Depth locations of the detected objects are found using CII blur-based analysis at the detected objects’ depth tubes. 

In this approach, the object depth localization operation would likely not be influenced by nearby objects, and it does not require sufficiently large objects and a sufficient depth distance between objects; thus, it is considerably more robust than examining the whole reconstructed plane to localize objects as done previously [28,30]. These characteristics, together with the use of a camera array, advance applicability in real-life scenes, as shown in the results.

A disadvantage of the method is that its first algorithmic stage relies on the 2D object detection capabilities of the instance segmentation. However, recent 2D object detection and segmentation methods using deep learning, such as Mask R-CNN, have very high accuracy performances, and are widely studied [40,41]. 

Future work will include depth-based tracking of objects in a 3D space and computational load improvement for real-time implementation. Furthermore, it is planned to create an integral-imaging image array database and combine machine learning-based 3D object detection methodology with the accurate object depth cues that our approach can produce for examining the accurate segmentation of objects in 3D space.

## Data Availability

Not applicable.
